# Implications of COVID-19 on urological laparoscopic surgery

**DOI:** 10.2217/fon-2020-0533

**Published:** 2020-06-27

**Authors:** Benjamin Condon, Thomas Whish-Wilson, Niall F Davis, Nathan Lawrentschuk

**Affiliations:** ^1^Peter MacCallum Cancer Centre, Division of Surgery, Melbourne, Australia; ^2^Department of Urology, E J Whitten Prostate Cancer Research Centre at Epworth, Victoria, Australia; ^3^Department of Surgery, University of Melbourne, Melbourne, Australia; ^4^Department of Urology, Austin Health, Melbourne, Australia; ^5^Department of Urology, St Vincent’s Hospital Melbourne, Melbourne, Australia; ^6^Department of Urology, Beaumont Hospital, Dublin, Ireland; ^7^Department of Surgery, The Royal College of Surgeons in Ireland, Dublin, Ireland; ^8^Department of Urology, Royal Melbourne Hospital, Melbourne, Australia

The turn of the decade will be remembered for the coronavirus disease 2019 (COVID-19) pandemic caused by the severe acute respiratory syndrome coronavirus 2 (SARS-CoV-2) that has spread throughout the globe. While the fight to contain the virus continues, the impact of the pandemic on healthcare systems around the world is becoming apparent. Of particular concern to urologists are data from early stages of the pandemic showing SARS-CoV-2 infection rates of 3% in postoperative patients undergoing urological procedures in the Republic of Ireland between 16 March 2020 and 1 May 2020 [1]. Hospitals have attempted to mitigate risk and conserve personal protective equipment (PPE) supplies by indefinitely postponing elective and nonessential surgery. Of the procedures unable to be delayed, the question of which surgical approach minimises risk to the patient and team: laparoscopic or open, is particularly topical.

When compared with open surgery, there are concerns that laparoscopic surgery confers a higher risk from an infection control perspective, of particular importance during the COVID-19 pandemic. This concern arises from the increased aerosolisation risk associated with desufflation of the pneumoperitoneum and exposure to surgical plumes, particularly with the use of ultrasonic scalpels and electrical surgical devices [2]. Desufflation causes a surgical plume to be created, which is a source of biological contamination containing blood cells, cell debris and potential viruses. This became particularly topical during the HIV epidemic, despite the fact HIV has never been isolated from a surgical plume. Recommendations asserted that surgical teams should exercise caution and use appropriate precautions to avoid all contact with all tissues and bodily fluid [2–4]. This notion has been echoed by Yu *et al.* when managing pneumoperitoneum in patients with or suspected of SARS-CoV-2 infection [1,5,6]. To date, there are no reports on SARS-CoV-2 aerosolisation by ablation, although CO_2_ circulating in the pneumoperitoneum may generate aerosols that contain SARS-CoV-2 virus [4]. This is particularly relevant when desufflating in the absence of an appropriate capture device. Indeed, the concerns regarding the increased risk of laparoscopic surgery led The Royal College of Surgeons of Edinburgh (Scotland) to recommend in their 27 March 2020 guidance to: “*consider laparoscopy only in selected individual cases where clinical benefit to the patient substantially exceeds the risk of potential viral transmission in that particular situation. Where nonoperative management is possible (such as for early appendicitis and acute cholecystitis) this should be implemented*” [5].

Applying this advice to urological procedures being undertaken during this pandemic is not straightforward. Table 1 highlights the conflicting guidelines and advice on managing urological laparoscopic surgery during the COVID-19 pandemic that have been published to date. Advances in both robotic, laparoscopic and laproendoscopic procedures has seen a significant reduction in the number of open surgeries being routinely performed by urologists. In particular, advances in ports used in transperineal and extraperitoneal robotic-assisted radical prostatectomy have led to a more stable pneumocavity and create a protective gas barrier within the ports themselves [7]. As discussed by Horstmann *et al.* who compared the AirSeal^®^ System valve-less trocar systems with standard Versaport™ Plus V2 Trocar system in patients undergoing robotic-assisted radical prostatectomy[7]. This brings into question the relevance of the concerns regarding desufflations increased aerosolisation risk, particularly in robotic surgery. Moreover, robotic surgery allows staff to be more remote from the patient and each other when compared with laparoscopic and open operations, facilitating better social distancing within the operating room [8]. Additionally, when compared with open procedures, laparoscopic approaches also generally allow improved social distancing between staff.

**Table 1.** Summary of recommendations for urological minimally invasive surgeries during the COVID-19 pandemic.

Study	Journal/guideline	Recommendations re lap surg in COVID-19	Ref.
Puliatti *et al.*	BJUI	Surgical intervention should be considered for emergencies, for example, high grade malignancies and unstable trauma patients. All surgeons should adopt appropriate PPE (Level 3)	[9]
Ficarra *et al.*	Minerva Urol Nefrol	Uro-oncological procedures divided into four categories: nondeferrable/urgent; semi nondeferrable; deferrable cancer surgery; replicable cancer surgery (radical prostatectomy considered as semi nondeferrable ; for intermediate to high risk patients) All benign or nononcological diseases delayed until the end of the COVID-19 emergency All outpatient procedures including biopsies should be delayed until post-COVID-19 emergency All surgery to be performed by experienced surgeons with a halt to clinical trials and new technologies	[10]
Nowroozi A & Amini E	Urol J	Limit urological procedures to emergencies and life-threatening cases. This includes delaying TURPs, radical cystectomy for high risk bladder cancer, radical prostatectomy for poorly differentiated PCa and radical nephrouretectomy	[11]
Mottrie A	EAUS	There is no current evidence to demonstrate COVID-19 in the CO_2_ plume created during MIS The concerns put forth by statements from SAGES and RCS may discourage surgeons from performing MIS surgery without adequate evidence Open surgery is not without viral transmission risk to the healthcare team and increases the burden on the healthcare system by increasing hospital bed occupancy with a longer length of stay MIS is superior to open surgery with regard to several patient outcomes across many disease states and conversion to open surgery represents a deviation from standard of care Because of the uncertainty surrounding COVID-19 in the CO_2_ plume, measures to decrease viral exposure to the surgical team should be performed	[12]
Porter *et al.*	BJU Int.	MIS /Laparoscopic surgery should be limited to planned urgent and emergency procedures Pre-operative COVID testing of patients if feasible Limit healthcare workers in room to essential personnel Surgical training should be limited to reduce time in operating room Social distancing within OT if able to Reduce surgical plume and pressure of the pneumoperitoneum Also summarised the ERUS position statement of key points (above)	[13]
Sobel *et al.*	Urology	Retrospective study of PPE use in different urologic procedures in an Italian single centre in March 2020. Percutaneous nephrostomy placement required the least PPE per procedure. Robotic-assisted urologic procedures consumed the most PPE per procedure	[14]
Narain *et al.*	Indian Journal of Cancer	Recommendation of deferring treatment of renal cell carcinoma from 3 to 6 months, except for patients with ongoing haematuria and/or inferior vena cava thrombus, which warrant immediate surgery Metastatic renal cell cancers should be started on targeted therapy Low grade nonmuscle invasive bladder cancers can be kept on active surveillance while high risk nonmuscle invasive bladder cancers and muscle invasive bladder cancers should be treated within 3 months Neoadjuvant chemotherapy should be avoided Management of low and intermediate risk prostate cancer can be deferred for 3–6 months while high risk prostate cancer patients can be initiated on neoadjuvant androgen deprivation therapy Patients with testicular tumors should undergo high inguinal orchiectomy and be treated according to stage without delay, with stage I patients being offered surveillance. Penile cancers should undergo penectomy, while clinically negative groins can be kept on surveillance Neoadjuvant chemotherapy should be avoided, and adjuvant therapy should be deferred	[15]
Ouzzane A & Colin P	Surgical Laparoscopy Endoscopy & Percutaneous Techniques	Surgical team protection includes a systematic screening of patients, wearing protection devices by all the operating staff and adequate management of aerosols. The risk of aerosol dispersal is particularly high during laparoscopic and robotic surgeries due to the interaction between circulating CO_2_ and surgical smoke that may contain small viral particles The use of integrated insufflation devices comprising smoke evacuation and filtration mode to decrease the risk of virus transmission	[16]
Moschovas *et al.*	EU Focus	Patients with a renal tumor ≥4 cm, demonstrate stage progression, growth kinetics >5 mm/yr or deterioration of their clinical condition should be considered for surgery if possible. Patients with bleeding tumors with a hemodynamic impact, tumors with renal vein and vena cava thrombus or large tumors causing compression of adjacent organs may be considered as surgical candidates during this COVID-19 period	[17]

Articles were identified via a Pubmed search, yielding 13 results. Four papers were excluded as they did not provide recommendations on how to approach urological surgeries amid the pandemic.

COVID-19: Coronavirus disease 2019; ERUS: EAU Robotic Urology Section; MIS: Minimally invasive surgery; OT: Operating Theatre; PCa: Prostate Cancer; PPE: Personal Protective Equipment; RCS: Royal College of Surgeon; SAGES: Society of American Gastrointestinal and Endoscopic Surgeon; TURP: Transurethral Resection of the Prostate.

We are still interrogating the safest ways to approach surgery in the COVID-19-era but there are certain aspects of the growing literature that have become clear. There is no evidence to confirm direct infection of members of a theatre team with the SARS-CoV-2 virus, or other viruses, from laparoscopic or open approach. Nevertheless, the ability of the SARS-CoV-2 virus to survive in certain environments has also been established. It remains viable in aerosols for 3 h with a half-life of 1.1–1.2 h [18]. It can survive on plastic and stainless steel for up to 72 h (half-life 5.6–6.8 h) [18]. As these materials are common within operating rooms, it is important to ensure adequate sterilization occurs both before and after surgical cases. There is also good evidence that contamination of said materials within an operating room can be contained by a negative pressure environment [19].

There are also questions when it comes to the fidelity of testing, with Lippi *et al.* documenting the limitations of the current reverse transcriptase (RT-PCR) method [20] . The RT-PCR test being used to confirm COVID-19 infections was shown to have a significantly high false negative rate of up to 30% with reliability depending on the pre-analytical handling of samples, selection of primers and quality of reagents and equipment [20]. Lippi *et al.* also asserts the limits of detection for the RT-PCR prevents the detection of positive patients with a low viral load, either in the initial phase of infection or following symptom relief [20]. As such, it is important to not be falsely reassured by a negative result. SARS-CoV-2 has been identified by Wang *et al.* in clinical specimens other than respiratory samples, including feces from 29% of patients, and in blood from a small number of patients. Importantly, for urologists, the virus has not been isolated in urine to date [21]. This is pertinent when urologists are considering natural orifice transluminal endoscopic surgery, specifically a transrectal hybrid natural orifice transluminal endoscopic surgery nephrectomy, which uses the rectum to gain access to the peritoneal cavity rather than the abdominal wall [22]. Despite being used infrequently, this approach to nephrectomy would lead to potential contamination of the access port and surgical field with SARS-CoV-2 in addition to colonic bacterial flora, potentially exposing the surgical team at increased risk of infection.

While concern regarding the COVID-19 pandemic remains ever present, it is important to understand the current gaps within the literature. An example of this ambiguity is the proposed utility in using Bacillus Calmette Guérin (BCG) vaccine to improve SARS-CoV-2 morbidity and mortality. Recent studies have shown countries without a recent or current universal BCG vaccination policy observed a slightly higher SARS-CoV-2 related morbidity and mortality than those countries that did [23]. This remains hypothetical at this stage without clear evidence. At present the most significant concern for urologists during this pandemic is to safely mitigate the risk of SARS-CoV-2 infection of both patient and the surgical team by considering the immediate need of a particular procedure, the approach and indeed even ports used to access the abdomen along with managing the surgical plume and having appropriate PPE and adjusting their planning accordingly.

We are learning more about COVID-19 daily, and as we look toward resumption of elective operating in Australia, it is important to consider which surgical procedures remain safest for both the patient and the surgical team alike. The absence of SARS-CoV-2 in urine provides urologists with some degree of reassurance when performing endoscopic work. However, when weighing the risk and benefits of an open, laparoscopic or robotic procedures, it remains a difficult decision. The theoretical risk of increased transmission posed by laparoscopic surgery needs to be considered. Nevertheless, the benefits of a minimally invasive surgical approach are long established, and these theoretical risks remain just that – theoretical. The increase in length of stay, in a high risk hospital environment after open surgery should also not be understated to a patient’s overall risk of infection [8]. We do know delaying a radical prostatectomy for prostate cancer for ≤12 months with no androgen deprivation therapy for all categories of localized prostate cancer is not associated with adverse postoperative outcomes [24]. This provides patients reassurance and allows urologists the opportunity to ensure appropriate time and caution is taken to protect both staff and patients during this pandemic.

## References

[1] McDermott A, O'Kelly J, de Barra E, Fitzpatrick F, Little DM, Davis NF Perioperative outcomes of urological surgery in patients with SARS-CoV-2 infection. Eur. Urol. 78(1), 118–120 (2020). 32425302 10.1016/j.eururo.2020.05.012 PMC7229918

[2] Collinson T, Hewett P, Hugh T, Padbury R, Maddern G Guidelines for safe surgery: open versus laparoscopic. a rapid review commissioned by RACS. The Royal Australian College of Surgeons. (2020). https://umbraco.surgeons.org/media/5214/2020-04-15-recommendations-on-safe-surgery-laparoscopic-vs-open.pdf

[3] Ott DE Prevention and management of laparoendoscopioc surgical complications. The Royal Australian College of Surgeons. 1 1–26 (2010).

[4] Eubanks S, Newman L, Lucas G Reduction of HIV transmission during laparoscopic procedures. Surg. Laparosc. Endosc. 3(1), 2–5 (1993). 8258065

[5] Griffin M, Alderson D, Taylor J, Melay K Updated Intercollegiate General Surgery Guidance on COVID-19 |25 March 2020. Royal College of Surgerons of Edinburgh, University of Edinburgh, Scotland (2020). www.rcseng.ac.uk/coronavirus/joint-guidance-for-surgeons-v2/

[6] Yu GY, Lou Z, Zhang W [Several suggestions of operation for colorectal cancer under the outbreak of corona virus disease 2019 in China]. Chinese J. Gastrointest. Surgy. 23(3), 208–211 (2020). 10.3760/cma.j.cn.441530-20200224-00074 32192295

[7] Horstmann M, Horton K, Kurz M, Padevit C, John H Prospective comparison between the AirSeal^®^ system valve-less Trocar and a standard Versaport™ plus V2 Trocar in robotic-assisted radical prostatectomy. J. Endourol. 27(5), 579–582 (2013). 23186377 10.1089/end.2012.0632

[8] Vigneswaran Y, Prachand VN, Posner MC, Matthews JB, Hussain M What is the appropriate use of laparoscopy over open procedures in the current COVID-19 climate? J. Gastrointest. Surg. (2020) (Epub ahead of print). 10.1007/s11605-020-04592-9 PMC7153352 32285338

[9] Puliatti S, Eissa A, Eissa R COVID-19 and urology: a comprehensive review of the literature. BJU International. 125(6), E7–E14 (2020). 10.1111/bju.15071 32249538

[10] Ficarra V, Novara G, Abrate A Urology practice during COVID-19 pandemic. Minerva Urol. Nefrol. 72(3), 369–375 (2020). 32202401 10.23736/S0393-2249.20.03846-1

[11] Nowroozi A, Amini E Urology practice in the time of COVID-19. Urol. J. 17(3), 326 (2020). 32207540 10.22037/uj.v0i0.6065

[12] Mottrie A ERUS (EAU robotic urology section) guidelines during COVID-19 emergency. 2020 https://uroweb.org/eau-robotic-urology-section-erus-guidelines-during-covid-19-emergency/

[13] Porter J, Blau E, Gharagozloo F Society of robotic surgery review: recommendations regarding the risk of COVID-19 transmission during minimally invasive surgery. BJU Int. (2020) (Epub ahead of print). 10.1111/bju.15105 PMC7267386 32383520

[14] Sobel D, Gn M, O'Rourke TK Personal protective equipment for common urologic procedures before and during the United States COVID-19 pandemic: a single institution study. Urology 141, 1–6 (2020). 32380154 10.1016/j.urology.2020.04.083 PMC7198432

[15] Narain T, Gautam G, Seth A Uro-oncology in times of COVID-19: the available evidence and recommendations in the Indian scenario. Indian J. Cancer 57(2), 129–138 (2020). 32445315 10.4103/ijc.IJC_356_20

[16] Ouzzane A, Colin P Cost-effective filtrating suction to evacuate surgical smoke in laparoscopic and robotic surgery during the COVID-19 pandemic. Surg. Laparosc. Endosc. Percutan Tech. (2020) (Epub ahead of print). 10.1097/SLE.0000000000000811 32487856

[17] Moschovas MC, Mazzone E, Puliatti S, Mottrie A, Patel V Selecting the most appropriate oncological treatment for patients with renal masses during the COVID-19 pandemic: recommendations from a referral center. Eur. Urol. Focus (2020) (Epub ahead of print). 10.1016/j.euf.2020.05.005 PMC7242959 32475783

[18] van Doremalen N, Bushmaker T, Morris DH Aerosol and surface stability of sars-cov-2 as compared with SARS-CoV-1. N. Engl. J. Med. 382(16), 1564–1567 (2020). 32182409 10.1056/NEJMc2004973 PMC7121658

[19] Park J, Yoo SY, Ko J-H Infection prevention measures for surgical procedures during a middle east respiratory syndrome outbreak in a tertiary care hospital in South Korea. Sci. Rep. 10(1), 325 (2020). 31941957 10.1038/s41598-019-57216-x PMC6962363

[20] Lippi G, Simundic AM, Plebani M Potential preanalytical and analytical vulnerabilities in the laboratory diagnosis of coronavirus disease 2019 (COVID-19). Clin. Chem. Lab. Med. 58(7), 1070–1076 (2020). 32172228 10.1515/cclm-2020-0285

[21] Wang W, Xu Y, Gao R Detection of SARS-CoV-2 in different types of clinical specimens. JAMA 323(18), 1843–1844 (2020). 10.1001/jama.2020.3786 PMC7066521 32159775

[22] Bazzi WM, Wagner O, Stroup SP Transrectal hybrid natural orifice transluminal endoscopic surgery (NOTES) nephrectomy in a porcine model. Urology 77(3), 518–523 (2011). 21376997 10.1016/j.urology.2010.10.057

[23] O'Connor E, Teh J, Kamat AM, Lawrentschuk N Bacillus calmette guérin (BCG) vaccination use in the fight against COVID-19 – what's old is new again? Fut. Oncol. 16(19), 1323–1325 (2020). 10.2217/fon-2020-0381 PMC7222530 32406253

[24] Sean Ong XR, Condon B, Bagguley D, Lawrentschuk N, Azad A, Murphy D Safety first: evidence for delay of radical prostatectomy without use of androgen deprivation therapy during COVID-19. Fut. Oncol. (2020) (Epub ahead of print). 10.2217/fon-2020-0388 PMC7226924 32407145

